# Evaluation of an Advanced Care Planning Training Program Incorporating Online Skills in Shared Decision Making: A Preintervention and Postintervention Comparative Study

**DOI:** 10.3390/healthcare11091356

**Published:** 2023-05-08

**Authors:** Yuko Goto, Hisayuki Miura

**Affiliations:** Department of Home Care and Regional Liaison Promotion, Hospital, National Center for Geriatrics and Gerontology, Obu 474-8511, Aichi, Japan; hmiura@ncgg.go.jp

**Keywords:** shared decision making, advance care planning, online skills training, new world kirkpatrick model, patient-centered care

## Abstract

Aim: This study evaluated an advanced care planning (ACP) training program incorporating online skills in shared decision making (SDM). Method: The New World Kirkpatrick Model was employed to assess the efficacy of the training program at four levels: reaction, learning, behavior, and results. Reaction measured the participants’ satisfaction and difficulty with the training program alongside the status of support received from workplaces engaging in ACP. Learning evaluated the changes in SDM skills. Behavior assessed the changes in the relationship between patients and healthcare professionals when the latter were involved in the SDM process. Results evaluated whether the participants were willing to participate in ACP educational programs as a facilitator and whether their motivation for continuous learning changed through throughout the training program. The relationships among patients, healthcare providers, and third-party roles were analyzed in SDM role-playing via structural equation modeling (SEM). Results: Between September 2020 and June 2022, 145 multidisciplinary participants completed the entirety of the training program. The most common responses to the training were “satisfied”, “slightly difficult”, and “I received some support from my workplace”. The SDM skills significantly improved from the first to the third workshop, evaluated using the Wilcoxon rank-sum test. In the first workshop, SDM was primarily performed by healthcare providers; however, in the third workshop, patient-centered SDM was adopted. Of the participants who completed the program, 63% intended to participate in future ACP educational programs as ACP education facilitators. Conclusion: This study ascertained the validity of this training.

## 1. Introduction

As the population ages, a Western concept of advanced care planning (ACP) has increasingly garnered attention in Japan [1,2,3]. Amid such circumstances, demand has been increasing for ACP programs that help the trainee understand ACP, acquire decision-making support skills, and achieve behavior modification conducive to practicing ACP. Moreover, healthcare professionals (HCPs) found it challenging to conduct face-to-face or group training sessions owing to the coronavirus disease 2019 (COVID-19) pandemic in 2020, which further highlighted the need to develop behavior-modifying training programs that allow the trainee to acquire decision-making support skills and learn ACP practices online.

The older adult population has been increasing globally, society has been evolving, and patients now have increasingly diverse values and options regarding treatment and therapy. ACP has been born out of the need to accommodate such changes and continues to evolve. Globally, ACP has been considered synonymous with completing advanced directives; however, recently, the core concept of ACP is regarded as a process of discussing medical and care matters with HCPs, such as physicians and trusted individuals. In addition, in around 2000, ACP was primarily performed for patients with tumor diseases; however, more recently, it is being performed for patients with various chronic diseases [4,5,6,7].

In Japan, the steady increase in the older population has finally led the public to recognize the importance of ACP in recent years. To disseminate the concept, the Ministry of Health, Labor, and Welfare nicknamed ACP “JINSEI KAIGI” (meaning life consultation) [8]. To make it better understood by the public, this “JINSEI KAIGI” is defined as “an effort to think beforehand about your desired medical treatment and healthcare choices in case of an emergency and repeatedly talk about and share those choices with your family and medical/healthcare professionals” [9]. In 2018, the “Guidelines for Decision Making about End-of-Life Care” [10] were revised to incorporate the concepts of ACP and shared decision making (SDM), thereby promoting the dissemination of the concept of end-of-life decision-making support. As stated above, demand has been growing for ACP training programs that help the trainee understand ACP, acquire decision-making support skills, and achieve behavior modification conducive to practicing ACP [11,12]. To meet this demand, the authors have been developing and evaluating face-to-face ACP training programs [13]. The outcomes of the ACP education program training have been evaluated primarily as trainee-related outcomes, including ACP knowledge, ability, and skills [14]; moreover, the in-person training program developed by us confirmed that the participants improved their SDM skills and changed their behavior with regard to ACP practice [13].

In Japan, multiple COVID-19 emergencies were declared in 2020 and 2021. Furthermore, a movement was implemented encouraging citizens to refrain from going out, thoroughly practicing “Social Distancing”, and avoid coming into contact with others. Thus, in-person training could not be held [15]. Therefore, the current COVID-19 pandemic has necessitated online (instead of in-person) ACP training programs that are equally effective.

The need to develop online training programs for HCPs has been recognized not only in Japan but also worldwide amid the COVID-19 pandemic, and various challenges have surfaced owing to the rapid digitalization of education [16,17,18,19,20,21].

With the increase in demand for online training owing to the COVID-19 pandemic, studies have concurrently reported training programs with demonstrated effects on behavior modification in HCPs [22,23]. SDM is a decision-support skill that emphasizes interactive communication between patients and professionals [24]. In palliative care (PC), which is conceptually similar to ACP, such ACP training programs use digital tools to encourage participants to hold conversations [25,26].

SDM is considered the pinnacle of patient-centered care [27] and contributes to decision making based on individual values for patients with various senses of worth [28]. Furthermore, SDM reportedly promoted dialogue between patients and professionals, filled an information gap regarding treatment and care options, improved patient satisfaction [29], improved adherence to and extended continued treatment [30,31], and decreased confrontations between patients and professionals, including litigation [32,33]. Meanwhile, patriarchal and familistic tendencies have been noted in Far East Asian countries, including Japan [34]. The familistic culture has been confirmed to reduce the degree of sharing in SDM [35]. This further highlights the importance of providing a thorough education to acquire SDM skills in Japan. SDM is a methodology for patient-centered decision support, and ACP is a process that helps the selection of treatment preferences and goals based on patient values [7]. With regard to the decision-support methods in the ACP process, instead of paternalistic or informed decisions, SDM alongside interaction between HCPs and patients centered on patient values constitutes the best choice [36].

Recently, we developed an online ACP training program incorporating skill training based on a previously developed face-to-face ACP training program [13] and implemented a training course. The purpose of this study was to evaluate this training program using the New World Kirkpatrick Model [37,38,39,40], which is used to evaluate medical and educational programs.

## 2. Materials and Methods

### 2.1. Study Design

This is a preintervention and postintervention comparative study. The intervention was educational and included three workshops. We used the SDM measurements that were immediately recorded after role-playing at the first and third workshops and the data obtained from the questionnaires related to the New World Kirkpatrick Model (except SDM measurement) that were administered after each workshop.

### 2.2. SDM Measurements Used

SDM-Q-9 Japanese [41], SDM-Q-Doc Japanese [42], SDM-C Japanese (patient) [43], and SDM-C Japanese (care staff) [43] are SDM measurements available in Japanese that were created based on SDM-Q-9 [44]/SDM-Q-Doc [45] developed by a team at the Department of Medical Psychology of the University of Hamburg-Eppendorf, Germany. These SDM measurements allow physicians, HCPs, and patients to evaluate processes to decide on treatment and care options using nine question items. These SDM measurements have sections that let the respondent write the reasons and results of their decision making. The respondent is asked to answer the nine questions on a 6-point Likert scale (ranging from “completely disagree” to “completely agree”).

### 2.3. Settings and Data Collection

The “Training on Shared Decision-making Competency in Advance Care Planning” program was based on a previously implemented in-person program [6]. However, as several participants were unfamiliar with online training, three short workshops were organized and incorporated into the online training program. In addition to the three online workshops, the online program included one preliminary e-learning session and two homework submissions over 6 months (Table 1).

The SDM measurement data were entered by the participants immediately after role-playing at the O1 and O3 workshop, which the participants took ~5 min to complete. These data were transcribed by training facilitators who listened to the measurement results of the participants. The training facilitators subsequently submitted the transcribed data to our researchers. The transcribed SDM measurement data were then converted into electronic data by information technicians who were not directly involved in this study.

Data from the questionnaires related to the New World Kirkpatrick Model (except SDM measurement) administered immediately after the three workshops (O1, O2, and O3, respectively) were collected online via an electronic questionnaire form. The organizers at the training sites provided the URL of the electronic questionnaire form to the participants and collected the questionnaire data online. The collected data were subsequently submitted to our researchers and used in this study.

### 2.4. Participants and Ethical Considerations

The data used in this study were collected from the participants of the “Training on Shared Decision-making Competency in Advance Care Planning” program that was sponsored and organized by six training sites between September 2020 and June 2022. The participants were HCPs in medicine, nursing, and welfare who were engaged in patients’ decision making in the clinical setting and potential future leaders in promoting ACP. They were recruited at each training site, which were hospitals and local governments in medium-sized cities and rural areas.

A common inclusion criterion for trainees is that participants must be qualified professionals involved in medical, nursing, and social welfare. The exclusion criterion includes those who do not belong to a clinical setting practicing ACP.

The reason for the inclusion criterion was that this training was intended for those who had received basic training in clinical ethics to practice ACP. The reason for the exclusion criterion was that as this training program was aimed at practicing ACP in clinical settings, the trainees had to be limited to those who worked in a field where ACP could be applied. At these six training sites, both physicians supporting decision making in treatment and healthcare specialists supporting decision making in healthcare participated in the workshops.

Each of the six training sites had its own rules for enrolling participants. Some sites recruited HCPs from within as well as outside their organization, whereas others recruited HCPs in specific sectors, such as PC. Before the workshops, the participants were informed that the data entered into worksheets during the workshops and the questionnaire data collected after the workshops would be used for evaluating the training program.

In this study, two data types were collected from each participant and analyzed. The first was the data on SDM measurements that were registered during the workshops. The second was the data on the questionnaire results that were written shortly after the three workshops. All data were anonymous.

The training was provided as an education rather than as research. To evaluate the quality of this program, we informed the sites that the program would be evaluated using the training data after the completion of the training and confirmed their intention to participate as training sites. The workshop and questionnaire data of the participants during this training were collected by the six training sites and training facilitators and shared with our researchers after removing personally identifiable information. The participants received an explanation for the use of the workshop data submitted by the participants before every workshop for program evaluation. During nearly 8 months, including the preparation period for the recruitment of the trainees, the results of the analysis of the training data were explained to the trainees and training site personnel during the training sessions.

Before implementation, this study was subjected to strict conflict of interest and research ethics reviews by the National Center for Geriatrics and Gerontology: IRB Approval Code and Name of the Institution: Approval code: no. 1585 (5 April 2022) from the National Center for Geriatrics and Gerontology.

### 2.5. Evaluation Framework

This training program was evaluated using the New World Kirkpatrick Model [37,38,39,40], which has been applied to various adult education programs, such as medical education programs [46,47,48] and nursing education programs [49,50,51]. This model is structured to evaluate educational programs using four levels, as follows: Level 1 concerning reaction, Level 2, learning, Level 3, behavior, and Level 4, results. In this study, the evaluation items were training satisfaction/difficulty and support from the participants’ workplace in practicing ACP (Level 1), SDM skills (Level 2), changes in relationships between patients and professionals in SDM (Level 3), and willingness to participate in future ACP training as an educational assistant for continued learning (Level 4). The entirety of this training program was evaluated by visualizing how this training program affected the continued learning needs of the participant.

### 2.6. Statistical Analyses

From the O1 questionnaire results, the characteristics of the participants were summarized, and the training satisfaction/difficulty and support from the participants’ workplace in practicing ACP were itemized. The answers were returned on a 6-point Likert scale.

The data on SDM skills collected by the SDM measurements were calculated with “completely disagree” as 0 points and “completely agree” as 5 points. The 45-point scores were then multiplied by 20/9 to convert them into 100-point scores and summarized into descriptive statistics. For SDM skills, the Wilcoxon rank-sum test calculated differences between O1 and O3.

The required sample size was calculated to be 67 for both O1 and O3 by the Wilcoxon rank-sum test (α = 0.05, power of test = 0.8, two-sided test, and the effective dose was set moderately at d = 0.5).

Changes in relationships between patients and professionals in SDM were analyzed by structural equation modeling (SEM) to confirm conceptual structures. The score from SDM1 to SDM9 is divided into the patient, provider, and third-party roles. The goodness of fit of the SEM was considered high when χ^2^ value (*p* > 0.05), the goodness of fit index (GFI) ≥ 0.9, the adjusted goodness of appropriate index (AGFI) ≥ 0.9, root-mean-square error of approximation (RMSEA) ≤ 0.05, and comparative fit index (CFI) ≥ 0.95.

Additionally, from the O1, O2, and O3 questionnaire results, changes in the participants’ perceptions were analyzed via SEM to confirm conceptual structures. The goodness of fit of the SEM was considered high when χ^2^ value (*p* > 0.05), GFI ≥ 0.9, AGFI ≥ 0.9, RMSEA ≤ 0.05, and CFI ≥ 0.95.

IBM SPSS Statistics 29 and IBM SPSS Amos Graphics 29 software (IBM Corp., Armonk, NY, USA) were used for statistical analyses.

## 3. Results

### 3.1. Overall Training Targets

This training program aimed to nurture human resources capable of practicing ACP by using SDM skills and providing education for promoting ACP. Accordingly, six training sites engaged in continued educational support publicly recruited participants who could offer education to promote ACP in local communities and organizations.

### 3.2. Participants’ Characteristics

At O1, the number of participants was 155, of whom 145 received a certificate of completion after finishing O3. The dropout rate was 6.5%.

A total of 153 questionnaire units were returned after O1. The participants’ professions (Table 2) and clinical experience years (Table 3) were itemized from these questionnaire results. By profession, the majority were nurses, accounting for about half of the participants, followed by physicians, medical social workers, care managers, and therapists. According to years of clinical experience, those with more than 25 years of experience accounted for 21%, and those with 15–19 years of experience and those with 20–24 years of experience each accounted for 20%. Overall, participants with varying years of clinical experience equally participated in this study.

### 3.3. Level 1 Reaction: Participants’ Satisfaction, Training Difficulty, and Support from the Participants’ Workplace in Practicing ACP

The reaction is evaluated at Level 1 of the New World Kirkpatrick Model. In this training program, the reaction evaluation items were the participants’ satisfaction after O1 (Table 4), training difficulty (Table 5), and support from the participants’ workplace in practicing ACP (Table 6).

Regarding the participants’ satisfaction, no respondents chose “not satisfied at all”. On the contrary, the most frequent and the second most frequent answers were “satisfied” and “very satisfied”, which were chosen by 55% and 23% of the respondents, respectively.

Regarding the training difficulty, the most frequent answer was “somewhat difficult”, chosen by 43%, which was followed by “difficult”, selected by 23%.

Regarding the support from the participants’ workplace in practicing ACP, the most frequent answer was “supported”, chosen by 35%, followed by “somewhat supported”, selected by 31%.

### 3.4. Level 2 Learning: SDM Skills

Learning is evaluated at Level 2 of the New World Kirkpatrick Model.

In this training, the learning results after O1 and O3 were itemized (Table 7).

The data on O1 and O3 were collected from 154 and 146 participants.

The Wilcoxon rank-sum test analyzed differences in the O1 and O3 results (Table 8).

The analysis showed that the SDM skills significantly improved in items SDM2, 3, 6, 8, and 9.

### 3.5. Level 3 Behavior: Changes in Relationships between Patients and Professionals in SDM

**Figure 1:**
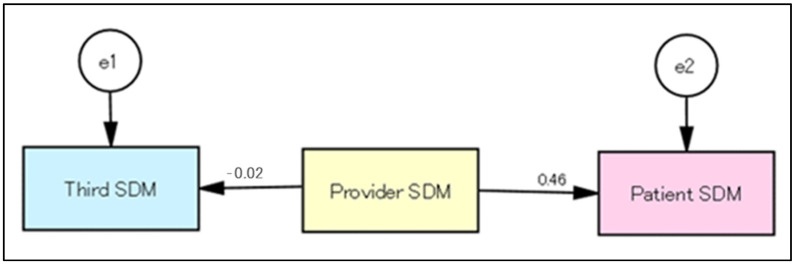
Relationship between the patient, provider, and third-party roles in role-playing at O1.

**Figure 2:**
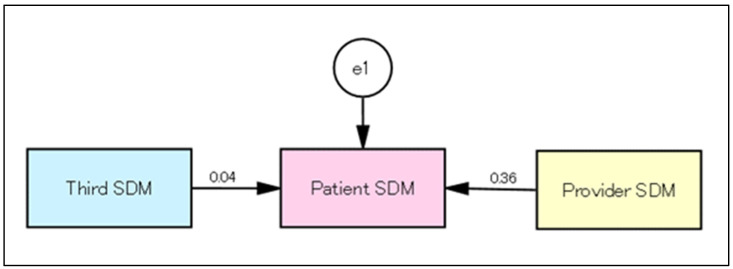
Relationship between the patient, provider, and third-party roles in role-playing at O3.

From the total score of SDM1 to SDM9, the relationships between patient, provider, and third-party roles in role-playing were analyzed by SEM. The results showed that the SDM at O1 was centered around the provider role (Figure 1). The model evaluation was χ^2^ = 0.031 (*p* = 0.861), GFI = 1.000, AGFI = 0.998, RMSEA = 0.000, and CFI = 1.000. In the SDM at O3, the relationship shifted to one centered around the patient role (Figure 2).

The model evaluation was χ^2^ = 0.005 (*p* = 0.946), GFI = 1.000, AGFI = 1.000, RMSEA = 0.000, and CFI = 1.000.

### 3.6. Level 4 Result: Willingness to Work as an ACP Education Facilitator and the Impact of This Training Program on the Participants’ Continued Learning Needs

Of the 145 participants who completed the program, 91 (63%) expressed willingness to participate in future online workshops and engage in educational activities for new trainees.

**Figure 3:**
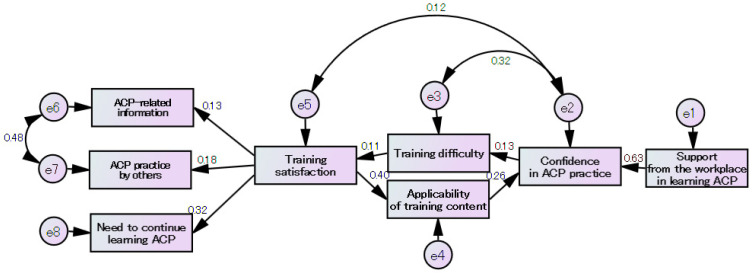
Impact of this training program on the participants’ continued learning needs.

Moreover, from the questionnaire results after O1, O2, and O3, the impact of this training program on the participants’ continued learning needs was visualized, and factors for causing continued learning needs in the participants were analyzed using SEM (Figure 3).

“Support from the workplace in learning ACP” led to “confidence in ACP practice”. Then, “training difficulty” led to “applicability of training content” and affected “training satisfaction”. Finally, the “need to continue learning ACP” was recognized, thereby increasing the awareness of “ACP practice by others” and “ACP-related information” listed in medical records, etc. The model’s goodness of fit was χ^2^ = 21.1 (*p* = 0.222), RMSEA = 0.023, and CFI = 0986.

## 4. Discussion

To the best of our knowledge, this was the first study in Japan that developed and evaluated an ACP training program incorporating online SDM skill training. The evaluation was performed up to Level 4 of the New World Kirkpatrick Model, and the results demonstrated the validity of this training program in nurturing human resources promoting ACP.

### 4.1. Evaluation by the New World Kirkpatrick Model at Different Levels

#### 4.1.1. Level 1 Reaction: Participants’ Satisfaction, Training Difficulty, and Support from the Participants’ Workplace in Practicing ACP

At Level 1, whether the content of the training program was acceptable to the participants was evaluated. Regarding the participants’ satisfaction, the most frequent answer was “satisfied”, and regarding the difficulty, the most frequent response was “somewhat difficult”. This indicated that the training program was perceived as moderately difficult by the participants and that the participants were satisfied with that difficulty level. Regarding the support from the participants’ workplace in practicing ACP, most participants responded that they were “supported”. Accordingly, it was highly probable that many participants worked in an environment where they could fully use the things they learned in this program. These results corroborated the valid training program in terms of reaction (Level 1).

#### 4.1.2. Level 2 Learning: SDM Skills

At Level 2, the improvement in SDM skills was evaluated. Among the three workshops, changes in the SDM measurement score were assessed at the first (O1) and third (O3) workshops, and significant improvement was noted in five of the nine items (SDM2, 3, 6, 8, and 9). This indicated that the SDM skill training significantly improved the SDM skills of the participants. Specifically, significant improvement in skills was observed in the following items: SDM2 “formulation of equality of partners”, SDM3 “presentation of treatment options”, SDM6 “identification of both parties’ understanding”, SDM8 “reaching a shared decision”, and SDM9 “arrangement of follow-up” [44]. However, regarding the skill items that did not achieve significant improvement in this skill training (SDM1 “disclosure that a decision needs to be made”, SDM4 “informing of the benefits and risks of the options”, SDM5 “investigation of patients’ understanding and expectations”, and SDM7 “negotiation”) [44], it is necessary to clarify challenges toward further qualitative improvements in the skills and have the participants undergo continued skill training. In particular, given that SDM4 “informing of the benefits and risks of the options” and SDM7 “negotiation” are essential factors in patient-centered medicine and care [52], further continued learning is required.

#### 4.1.3. Level 3 Behavior: Changes in Relationships between Patients and Professionals in SDM

The results of SDM in this training program revealed a shift in the relationship from the one centered around the specialist at O1 to the one centered around the patient at O3.

SDM is regarded as the pinnacle of patient-centered care [28], whose purpose is to support patients’ autonomy [53,54]. The intervention of this training program visualized the behavior modification of the participants in decision making. Herein, as the SDM measures were recorded immediately after each role-play, it was not possible to grasp how long this effect lasted. This is one of the limitations of this study. However, this suggested that the hypothesis that SDM is the pinnacle of patient-centered care could be similarly applied to the clinical setting in Japan.

#### 4.1.4. Level 4 Result: Willingness to Work as an ACP Education Facilitator and the Impact of This Training Program on the Participants’ Continued Learning Needs

The purpose of this training program was to nurture future leaders in promoting ACP. If a participant who has completed the program expresses willingness to work as a facilitator or educational staff in future sessions, they are highly likely to act toward specific goals. When this happens, the purpose of this training program (human resource nurturing) is considered to have been achieved.

Of the 145 participants, 91 (63%) expressed willingness, whereas the remaining 37% did not. In light of this result, there is room for consideration regarding the result evaluation (Level 4). Since the lack of willingness to participate in future training sessions does not necessarily mean that the person is not qualified as a leader promoting ACP, more adjustments will be needed in future research, including reconsidering the evaluation items.

Meanwhile, when the entirety of this training program was comprehensively evaluated, and the factors affecting the participants’ perception about continued learning needs were searched, it was revealed that “support from the participants’ workplace in practicing ACP” led to “confidence in ACP practice” and “training satisfaction” as the training program progressed, thus affecting continuous learning needs. This indicates that it is essential to encourage the understanding of the workplace in ACP practice to “nurture future leaders in promoting ACP”. A previous study identified a lack of cooperation and understanding in the workplace as barriers to ACP practice [6]. Another study indicated that a lack of support obstructs ACP practice owing to environmental factors, such as insufficient legal and medical service fee systems [55]. Therefore, approaches to change workplace understanding and environment are critical for producing leaders promoting ACP. Hopefully, the participants nurtured in this training program will rejuvenate ACP activities in the future.

This training program has obtained understanding and cooperation from the training sites sponsoring the training, including local core hospitals and medical associations. These training sites are taking the initiative in nurturing human resources. This framework may be conducive to supporting ACP-promoting leaders who completed this program in their continued learning needs.

### 4.2. Significance of Developing an ACP Training Program Incorporating Online SDM Skill Training in Japan

Japan has the world’s highest percentage of senior citizens, accounting for approximately 30% [56]. The Ministry of Health, Labor, and Welfare published the “Guidelines for medical and care decision-making processes at the end of life” [10], and the Japan Geriatric Society [57] and the Japanese Society for Dialysis Therapy [58] issued proposals and guidelines related to SDM and ACP. However, SDM skill training compatible with the characteristics of Japanese society is not domestically available. The mainstream of ACP training programs is primarily aimed at knowledge acquisition [59] and programs whose effectiveness has only been demonstrated for certain professions [60].

The major challenge in today’s Japan is the lack of opportunities for receiving SDM skill training. However, this effective online ACP training program, incorporating SDM skill training developed in this study, will provide opportunities for HCPs living in hard-to-access remote areas to participate in activities effectively. Furthermore, we believe this achievement is socially significant in today’s Japanese society.

Moreover, in many East Asian countries, including Japan, group decision making tends to be culturally more appreciated than individual decision making [61]. A preceding study indicated that the familism influenced by Confucianism weakens the SDM of patients with progressive cancer and clinicians engaged in end-of-life care [36]. Accommodating these conditions, some researchers have proposed Japanese versions of ACP definitions that promote family-centered decision making [62], showing a regressive trend contrary to patient-centered medical care. One of the factors behind this trend may be the lack of opportunities for learning SDM, which is regarded as the pinnacle of patient-centered medical care. Therefore, developing this effective ACP training program incorporating SDM skill training is highly significant for Japanese society.

### 4.3. Strengths and Limitations of the Study

Japan lags behind major Western countries in the availability of online infrastructure [63]. Numerous challenges have been pointed out in the educational setting for educators and students [64,65]. For example, one week before each workshop, training for online operations was provided to the medical institutions acting as study sites to prepare for online training management. This placed a heavier workload on them than face-to-face training management. Moreover, some participants had to cancel a course owing to network instability during the training. Since Japan is still developing online infrastructure, improvements are needed to disseminate online education widely. Presently, this is a limitation of online training in Japan.

Meanwhile, since the training sites in this study agreed to support the participants’ ACP practice after repeated coordination with our researchers, the participants recruited by these training sites may have been biased toward behavior modification, as observed in this study. However, as ACP is not well recognized in Japan [55,66], many organizations remain uncooperative toward ACP practice. Therefore, in the future, besides nurturing human resources, efforts to promote the understanding of ACP for such organizations should be made simultaneously. Otherwise, this training program will not function effectively in Japan. This is another limitation of this study.

## 5. Conclusions

This study developed an ACP training program incorporating online SDM skill training as an educational program for nurturing leaders promoting ACP. The New World Kirkpatrick Model ascertained the validity of this training program.

## Figures and Tables

**Table 1:** Online educational programs and data collection points.

Course	Training Day (Total Learning Time)	Outline	Collected Data	Observation Number
E-learning	From 1 month before until the first workshop (~4–7 h)	Acquisition of basic knowledge related to SDM and ACP		
First online workshop		Acquisition of applied knowledge related to SDM and ACP SDM communication skills training (role-play) This role-play was a simulation	Number of prelearning completion certificates submitted Results of the questionnaires administered after the workshop Data of the SDM measurements	O1
First practice and report-making session at home	Arbitrary	Practice in participants’ workplace		
Second online workshop	1 month after the first workshop (3.5 h)	Acquisition of applied knowledge related to interprofessional SDM and goals-of-care discussion Team building session for ACP via discussion between professionals (group discussion)	Results of questionnaires after the workshop Date of the group’s SDM measurements	O2
Second report-making session at home	Arbitrary	Practice at the participants’ workplace		
Third online workshop	3 months after the second workshop (4 h)	Feedback of own practice SDM communication skills training (role-play) This role-play was a simulation. Making and announcing activity plans (small group discussion)	Number of practice reports submitted Results of the questionnaires administered after the workshop Data of the SDM measurements	O3

ACP, advance care planning; SDM, shared decision making.

**Table 2:** Professions of the participants at O1 (*n* = 153).

Profession	Number	Percentage (%)
Nurse	75	49
Physician	24	16
Medical social worker	16	10
Care manager	11	7
Therapist	9	6
Counselor	6	4
Others	12	8
Total	153	100

**Table 3:** Clinical experience years of the participants at O1 (*n* = 153).

Clinical Experience Years as a Professional	Number	Percentage (%)
<5 years	20	13
5–9 years	22	15
10–14 years	17	11
15–19 years	31	20
20–24 years	31	20
≥25 years	32	21
Total	153	100

**Table 4:** The degree of satisfaction perceived by the participants after O1 (*n* = 153).

Degree of Satisfaction	Number	Percentage (%)
Not satisfied at all	0	0
Not satisfied	0	0
Somewhat not satisfied	4	3
Somewhat satisfied	29	18
Satisfied	83	55
Very satisfied	36	23
No answer	1	1
Total	153	100

**Table 5:** Training difficulty perceived by the participants after O1 (*n* = 153).

Training Difficulty	Number	Percentage (%)
Very difficult	15	10
Difficult	35	23
Somewhat difficult	67	43
Somewhat easy	28	19
Easy	5	3
Very easy	3	2
Total	153	100

**Table 6:** Support from the O1 participants’ workplace in practicing ACP (*n* = 153).

Support from the Participants’ Workplace in Practicing ACP	Number	Percentage (%)
Not supported at all	4	3
Not supported	10	6
Somewhat not supported	9	6
Somewhat supported	47	31
Supported	54	35
Fully supported	28	18
N/A	1	1
Total	153	100

**Table 7:** Descriptive statistics of nine SDM measurement items after O1 and O3.

		Median	Mean	SD	Minimum	Maximum
The first workshop (O1)	SDM1	8.89	7.89	2.424	0	11
*n* = 154	SDM2	6.67	7.33	2.666	0	11
	SDM3	6.67	7.23	2.635	0	11
	SDM4	6.67	5.74	2.949	0	11
	SDM5	6.67	7.27	2.414	2	11
	SDM6	8.89	7.75	2.877	0	11
	SDM7	6.67	5.79	2.658	0	11
	SDM8	6.67	7.53	2.595	0	11
	SDM9	8.89	7.91	2.342	0	11
Third Workshop (O3)	SDM1	8.89	8.3	2.188	0	11
*n* = 146	SDM2	8.89	8.1	2.226	2	11
	SDM3	8.89	7.93	2.516	0	11
	SDM4	6.67	5.92	2.703	0	11
	SDM5	8.89	7.79	2.493	0	11
	SDM6	8.89	8.57	2.392	0	11
	SDM7	6.67	5.98	2.719	0	11
	SDM8	8.89	8.08	2.569	0	11
	SDM9	8.89	8.45	2.401	0	11

**Table 8:** Results of Wilcoxon rank-sum test on differences between O1 and O3.

	SDM1	SDM2	SDM3	SDM4	SDM5	SDM6	SDM7	SDM8	SDM9
Wilcoxon W	22,271.5	21,320.5	21,447	22,841	21,865	21,413.5	22,842	21,623	21,610
Z	−1.259	−2.565	−2.38	−0.461	−1.816	−2.437	−0.462	−2.154	−2.194
*p*	0.208	0.01 *	0.017 *	0.645	0.069	0.015 *	0.644	0.031 *	0.028 *

* *p* < 0.05.

## Data Availability

The data used to support the findings of this study are available from the corresponding author upon request.
