# Correction: Goto, Y.; Miura, H. Evaluation of an Advanced Care Planning Training Program Incorporating Online Skills in Shared Decision Making: A Preintervention and Postintervention Comparative Study. *Healthcare* 2023, *11*, 1356

**DOI:** 10.3390/healthcare11152138

**Published:** 2023-07-26

**Authors:** Yuko Goto, Hisayuki Miura

**Affiliations:** Department of Home Care and Regional Liaison Promotion, Hospital, National Center for Geriatrics and Gerontology, Obu 474-8511, Aichi, Japan

In the original publication [1], there were mistakes in Tables 3–5 as published. 

The number of the ‘15–19 years’ row in Table 3 was 31 instead of 30. The number and percentage for the ‘Not satisfied’ row in Table 4 were 0 instead of 1. The number of the ‘Satisfied’ row in Table 4 was 83 instead of 84. A ‘No answer’ row in Table 4 was added, with the number and percentage of 1. The number in the ‘Somewhat easy’ row in Table 5 was 28 instead of 29. The corrected Table 3, Table 4 and Table 5 appear below. 

The authors state that the scientific conclusions are unaffected. This correction was approved by the Academic Editor. The original publication has also been updated. 

## Figures and Tables

**Table 3:** Clinical experience years of the participants at O1 (*n* = 153).

Clinical Experience Years as a Professional	Number	Percentage (%)
<5 years	20	13
5–9 years	22	15
10–14 years	17	11
15–19 years	31	20
20–24 years	31	20
≥25 years	32	21
Total	153	100

**Table 4:** The degree of satisfaction perceived by the participants after O1 (*n* = 153).

Degree of Satisfaction	Number	Percentage (%)
Not satisfied at all	0	0
Not satisfied	0	0
Somewhat not satisfied	4	3
Somewhat satisfied	29	18
Satisfied	83	55
Very satisfied	36	23
No answer	1	1
Total	153	100

**Table 5:** Training difficulty perceived by the participants after O1 (*n* = 153).

Training Difficulty	Number	Percentage (%)
Very difficult	15	10
Difficult	35	23
Somewhat difficult	67	43
Somewhat easy	28	19
Easy	5	3
Very easy	3	2
Total	153	100
